# Beyond euploidy: the synergistic value of blastocyst morphology in predicting ongoing implantation in advanced maternal age women

**DOI:** 10.1530/RAF-26-0026

**Published:** 2026-05-18

**Authors:** Chatsarun Srimoung, Artitaya Singwongsa, Chonthicha Satirapod, Matchuporn Sukprasert, Makaramas Anantaburana

**Affiliations:** ^1^ Department of Obstetrics and Gynaecology, Faculty of Medicine, Ramathibodi Hospital, Mahidol University, Bangkok, Thailand; ^2^ Reproductive Endocrinology and Infertility Unit, Department of Obstetrics and Gynaecology, Faculty of Medicine, Ramathibodi Hospital, Mahidol University, Bangkok, Thailand

**Keywords:** advanced maternal age, blastocyst morphology, euploidy, ongoing implantation rate

## Abstract

**Abstract:**

This single-centre retrospective study evaluated whether blastocyst morphology remains an independent predictor of euploidy and implantation rates in an advanced maternal age (AMA) population after controlling for chromosomal status. Analysing 1,067 biopsied blastocysts from 308 IVF cycles (269 patients) between October 2021 and December 2022, embryos were graded via the Gardner and Schoolcraft system and categorised into Excellent, Good, Average, and Poor groups. The overall euploidy rate was 33.62%, with blastocysts of the Excellent group exhibiting a significantly higher euploidy rate compared to the Good, Average, and Poor groups (65.38 vs 54.74, 31.46, and 18.79%, respectively; *P* < 0.001). Logistic regression confirmed that Excellent/Good (odds ratio (OR): 5.60; 95% CI: 4.07–7.71) and Average (OR: 1.98; 95% CI: 1.40–2.81) embryos were significantly more likely to be euploid than Poor embryos. Among confirmed euploid transfers, morphological grade significantly impacted clinical outcomes: Excellent/Good grades yielded an implantation rate of 52.38%, whereas Poor grades yielded only 23.81% (*P* < 0.05). Multivariate analysis verified that morphological grade is a significant prognostic factor for implantation, independent of ploidy status. In conclusion, euploidy does not equalise implantation potential across all morphological grades in older women. Thus, blastocyst morphology serves as a critical secondary selection tool, suggesting that Poor-grade euploid embryos may harbour non-chromosomal compromises that affect developmental competence.

**Lay summary:**

When patients undergo IVF, they often use genetic testing (PGT-A) to select embryos with the correct number of chromosomes, known as ‘euploid’ embryos. While it is often suggested that genetic normalcy is the most critical factor for success, the importance of an embryo’s physical appearance (morphology) in these cases remains a subject of debate. Our study investigated this in women aged 35 or older by analysing over 1,000 embryos and found that, even among those with normal genetics, morphology still plays a vital role. Specifically, genetically normal embryos with poor physical appearances had significantly lower chances of successful implantation compared to high-quality ones. We discovered that the quality of the ‘inner cell mass’, the part that becomes the baby, is a particularly strong predictor of success. These findings suggest that, for older patients, choosing an embryo based on both genetics and appearance is essential for the best pregnancy outcome.

## Introduction

Advanced maternal age (AMA) has become a predominant challenge in modern reproductive medicine, directly contributing to increased rates of embryonic aneuploidy and implantation failure (Sun *et al.* 2019, Shen *et al.* 2024). To mitigate these risks, preimplantation genetic testing for aneuploidy (PGT-A) has been widely adopted as the gold standard for embryo selection, aiming to improve live birth rates and reduce miscarriage risks by selecting only chromosomally normal blastocysts for transfer (Zaat *et al.* 2021). Consequently, the clinical focus has shifted heavily towards chromosomal status, leading to a prevalent ‘euploid-centric’ model of care (Gardner & Schoolcraft 1998, Gardner *et al.* 2015, Viotti 2020).

However, the prioritisation of PGT-A has sparked a significant debate regarding the remaining value of traditional morphological grading. Historically, blastocyst morphology based on the Gardner & Schoolcraft system was the primary predictor of viability (Gardner & Schoolcraft 1998, Gardner *et al.* 2015). Yet, recent studies suggest that once an embryo is confirmed euploid, its morphological grade may have little to no impact on implantation potential (Capalbo *et al.* 2014, Zhang *et al.* 2025). This has led some clinicians to deprioritise morphological assessment, operating under the assumption that an ‘euploid is a euploid’, regardless of its structural quality (Minasi *et al.* 2016, Li *et al.* 2022).

Despite this shifting paradigm, relying solely on chromosomal screening may be insufficient, particularly for the AMA population. Euploidy confirms chromosomal segregation but does not account for the embryo’s metabolic health, mitochondrial function, or structural integrity – factors often compromised in oocytes from older women (Fragouli *et al.* 2015, May-Panloup *et al.* 2016). Therefore, it is biologically plausible that a euploid embryo with poor morphology (e.g. sparse inner cell mass (ICM) or loose trophectoderm (TE)) may still harbour functional deficits that limit implantation. Evidence specifically clarifying the interplay between detailed morphological parameters (ICM vs TE) and implantation outcomes in euploid embryos from older women remain limited and conflicting.

This study aims to investigate whether blastocyst morphology remains an independent prognostic factor for ongoing implantation rate (OIR) among confirmed euploid embryos. Unlike previous studies that largely focus on young, good-prognosis patients, we specifically analyse a cohort of patients with AMA to determine whether morphology acts as a critical secondary filter. Furthermore, we seek to dissect which specific morphological components, degree of expansion, ICM, or TE, hold the highest predictive value for success when chromosomal status is controlled.

## Materials and methods

### Study design and population

This was a retrospective, cross-sectional study using medical record data from 269 patients who underwent 308 IVF cycles with PGT-A on blastocysts performed between October 2021 and December 2022 at Ramathibodi Hospital. This study included embryos from women aged between 20 and 45 years old who were undergoing both intracytoplasmic sperm injection (ICSI) and PGT-A of the blastocyst. The exclusion criteria were those with data loss and inconclusive PGT-A results.

### Ovarian stimulation protocol

Gonadotropin-releasing hormone (GnRH) antagonist and agonist protocols were used in this study. Gonadotropin doses were formulated based on the patient’s age and ovarian reserve, including antral follicle count (AFC), anti-Müllerian hormone levels, basal follicle-stimulating hormone levels, and prior response to stimulation. A transvaginal ultrasound was performed to monitor follicular response to stimulation, and gonadotropin doses were adjusted accordingly. Final oocyte maturation was triggered with 0.2 mg of diphereline (Ipsen Pharma Biotech, France) or 250 μg of recombinant human chorionic gonadotropin (hCG;Ovidrel, Merck, USA) with or without a GnRH agonist when the mean diameter of at least two follicles was 16 mm. Ultrasound-guided oocyte retrieval under conscious sedation was performed 35.5–36.5 h after the trigger.

### Laboratory protocols

Fertilisation was performed in all meiosis II oocytes 4 h after oocyte pick-up by ICSI. The fertilisation check was then carried out 16–18 h after ICSI. All embryos were cultured to the blastocyst stage in an Origio sequential medium (CooperSurgical, USA) with sterile paraffin oil-covered media. The culture system was set at 37°C, 6% CO_2_, and 5% O_2_.

### Embryo morphologic grading and biopsy

All blastocysts were evaluated for morphology on the 5th day using the Gardner and Schoolcraft grading system, based on the degree of expansion, ICM, and TE (Gardner & Schoolcraft 1998), by two experienced embryologists to ensure consistency.

The degree of expansion grading included:

- The blastocele is less than half the volume of the embryo.
- The blastocele is greater than or equal to half of the volume of the embryo.
- The blastocele completely fills the embryo.
- The blastocele volume is more significant than that of the early embryo, and the zona pellucida is thinning.
- The trophectoderm has started to herniate through the zona pellucida (hatching).
- The blastocyst has completely escaped from the zona pellucida (hatched).

The ICM grading included:

- Tightly packed with many cells
- Loosely grouped with several cells
- Having very few cells

The TE grading included:

- Many cells forming a tightly knit epithelium
- Few cells
- Very few cells forming a loose epithelium

The embryo grades were categorised into four quality groups:

Excellent: 6AA, 6AB, 6BA, 5AA, 5AB, 5BA.

Good: 6BB, 6AC, 6CA, 6BC, 6CB, 6CC, 5BB, 5AC, 5CA, 5BC, 5CB, 5CC, 4AA, 4AB, 4BA, 4BB, 4AC, 4CA, 4BC.

Average: 4CB, 4CC, 3AA, 3AB, 4BA, 3BB, 3AC, 3CA, 3BC, 3CB, 3CC.

Poor: 2AA, 2AB, 2BA, 2BB, 2AC, 2CA, 2BC, 2CB, 2CC, 1AA, 1AB, 1BA, 1BB, 1AC, 1CA, 1BC, 1CB, 1CC.

Then, the embryos were selected for biopsy. Due to economic considerations, embryos of lower quality were generally not chosen for the trophectoderm biopsy unless they were the only viable options within the couple’s embryo cohort. For TE biopsy, the dishes contained nine drops of culture media, each measuring 20–30 mL. On day 5, embryos were biopsied using an Eclipse Ti-heated microscope (TE-2000; Nikon, Japan) equipped with a Narishige micromanipulator (NT-88;Narishige, Japan). The blastocysts were held in place with a pipette on the left side while the zona pellucida was perforated using laser pulses from a ZILOS-tk noncontact laser (Hamilton Thorne Biosciences, USA). A suitable side biopsy pipette was then used to gently suction TE cells through the zona pellucida perforation, allowing them to be separated. The biopsied TE cells were washed with 1% PVP in D-phosphate-buffered saline (D-PBS; Ca^2+^ and Mg^2+^ removed) and placed in RNase–DNase-free 0.2 mL polymerase chain reaction (PCR) tubes for preimplantation genetic testing for aneuploidy (PGT-A). Next-generation sequencing (NGS) using the Illumina MiSeq® sequencing platform (Illumina Inc., USA)and the Thermo Fisher Scientific Inc. (USA) platform was employed to diagnose embryos as euploid, aneuploid, or mosaicism. All blastocysts were vitrified 1 h after biopsy. In subsequent cycles, the frozen euploid embryos were thawed and used for single-embryo transfers.

### Outcome measures

The primary outcome of this study was the euploidy rate in day 5 blastocysts. The secondary outcome evaluated the correlation between blastocyst morphologic grading and euploidy rates, and compared morphologic parameters. The study also assessed the association between blastocyst morphological grade and euploid rates within different age groups. Moreover, the OIR between different morphologic grades and age groups was also evaluated for the pregnancy outcomes.

### Statistical analysis

Continuous data were presented as mean ± standard deviation when the data were normally distributed, and the median was used for variables that did not show normality. Data were compared using Student’s *t*-tests or Mann–Whitney *U* tests. Categorical data were presented as counts and percentages, and the chi-square test was used for statistical analysis. Logistic regression analysis was used to investigate the effect of blastocyst morphology, morphological parameters, euploidy rate, and OIR. Variables considered potential confounders were selected based on clinical relevance and prior literature, including maternal age, BMI, duration of infertility, infertility diagnosis, type of infertility, parity, indication for PGT-A, and AFC. Variables with *P* < 0.10 in univariate analysis were subsequently entered into the multivariate logistic regression model. Odds ratios (ORs) and 95% confidence intervals (CIs) were calculated. The level of statistical significance was set to *P* < 0.05. All statistical analyses were performed using Stata 18.0 (Stata; USA).

## Results

### Study population and embryological outcomes

**Figure 1:**
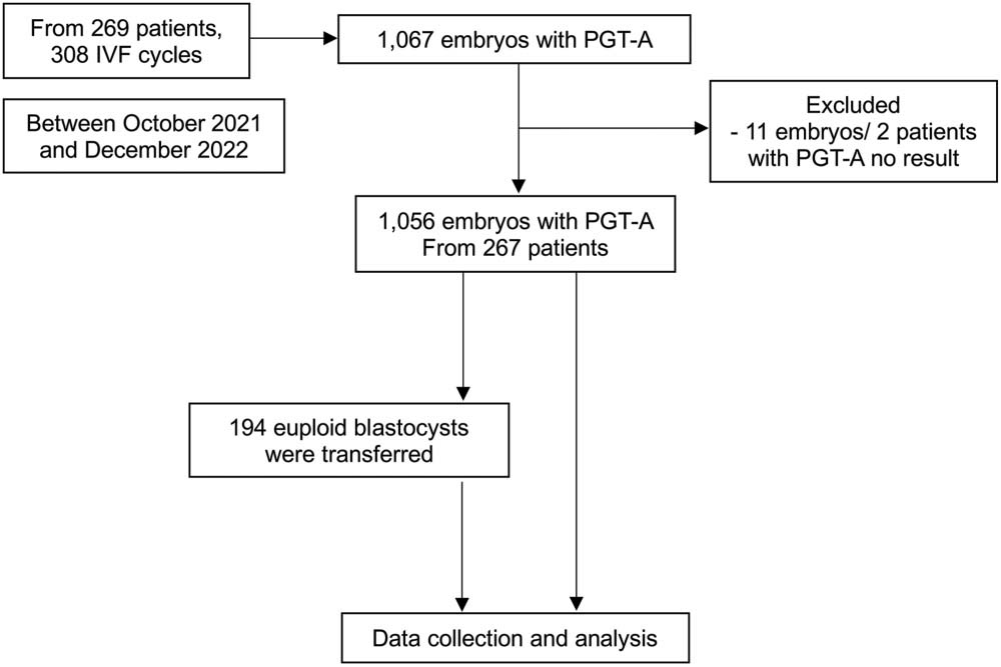
Protocol flow chart of this study.

**Figure 2:**
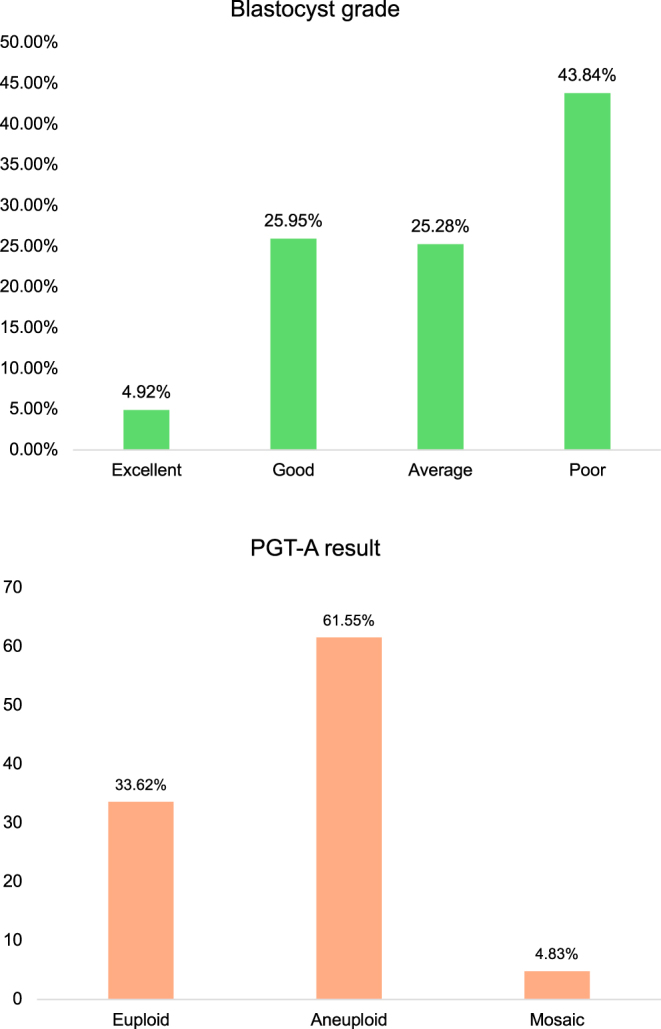
Blastocyst morphology grading and PGT-A result.

From 308 IVF cycles (269 patients), 1,067 blastocysts were biopsied. After excluding 11 inconclusive PGT-A results, 1,056 blastocysts remained for analysis (Fig. 1). The mean maternal and paternal ages were 38.25 ± 2.88 and 39.74 ± 5.20 years, respectively (Table 1). AMA (≥35 years) was the primary indication for PGT-A (96.63%). Laboratory outcomes showed a median of 11 retrieved oocytes and 5 blastocysts per cycle. Morphology distribution (Fig. 2) was Poor (43.84%), Good (25.95%), Average (25.28%), and Excellent (4.92%).

**Table 1:** Baseline characteristics of patients and embryological outcomes in the IVF cycle. Data are presented as *n* (%), mean ± SD, or median (IQR).

Parameters	Values
*n*	267
Female age (years)	38.25 ± 2.88
Age group	
<35 years	14 (5.24)
35–37 years	96 (35.96)
38–41 years	104 (38.95)
≥41 years	53 (19.85)
Male age (years)	39.74 ± 5.20
BMI (kg/m^2^)	22.63 ± 3.72
Infertility types	
Primary	161 (60.30)
Secondary	91 (34.08)
For PGT-M	15 (5.62)
Duration of infertility (years)	3.77 ± 3.10
Number of oocytes retrieved/cycle	11 (8, 16)
Number of available blastocysts	5 (3, 7)
Number of blastocysts biopsied	50.00 (33.33, 62.50)
Indication for PGT-A	
Male or female genetic abnormality	15 (5.62)
Previous child with genetic abnormality	18 (6.74)
Stem cell transplant from HLA matched	2 (0.75)
≥2 unexplained recurrent pregnancy losses	15 (5.62)
Advanced maternal age (≥35 years)	258 (96.63)
≥2 unsuccessful ART treatments	27 (10.11)

### Predictors of euploidy

**Figure 3:**
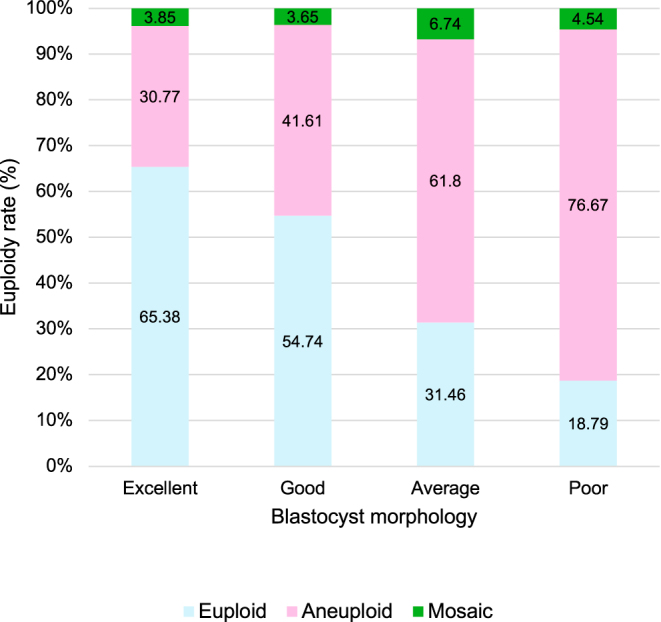
Correlation between blastocyst morphology and euploidy rate.

Total euploid embryos were 355. The overall euploidy rate was 33.62% (Fig. 2). Higher morphological grades correlated with significantly higher euploidy rates: 65.38, 54.74, 31.46, and 18.79% for Excellent, Good, Average, and Poor grades, respectively (*P* < 0.001; Fig. 3). Multivariate analysis confirmed that blastocyst grade, TE quality, and maternal age were independent predictors of euploidy (Table 2). Specifically, Excellent/Good embryos (OR: 3.75) and TE grades A/B (OR: 2.34 and 1.77) showed higher euploidy odds than Poor/grade C counterparts. Conversely, maternal age ≥38 years significantly reduced euploidy odds (*P* < 0.001).

**Table 2:** Univariate and multivariate logistic regression analysis of the association between blastocyst morphology, age group, and euploidy rate.

Variable	Univariate analysis		Multivariate analysis	
	OR (95% CI)	*P* value	OR (95% CI)	*P* value
Blastocyst grade				
Excellent and Good	5.60 (4.07–7.71)	0.00	3.75 (2.56–5.50)	0.00
Average	1.98 (1.40–2.81)	0.21	1.47 (1.00–2.14)	0.05
Poor	1.00	-	1.00	-
Degree of expansion				
5, 6	9.08 (4.69–17.59)	0.00	-	-
4	5.54 (3.69–8.32)	0.00	-	-
3	2.23 (1.46–3.40)	0.00	-	-
2	1.28 (0.80–2.36)	0.30	-	-
1	1.00	-	-	-
ICM				
A	9.13 (4.95–16.86)	0.00	-	-
B	2.99 (2.12–4.21)	0.00	-	-
C	1.00	-	-	-
TE				
A	8.21 (4.56–14.77)	0.00	2.34 (1.19–4.63)	0.01
B	3.03 (2.24–4.09)	0.00	1.77 (1.25–2.50)	0.00
C	1.00	-	1.00	-
Age group (years)				
<35	1.00	-	1.00	-
35–37	0.76 (0.43–1.35)	0.35	0.93 (0.51–1.70)	0.81
38–40	0.32 (0.18–0.56)	0.00	0.40 (0.22–0.73)	0.00
≥41	0.12 (0.06–0.24)	0.00	0.16 (0.08–0.34)	0.00
BMI (kg/m^2^)				
<18.5	0.94 (0.58–1.50)	0.78	-	-
18.5–24.9	1.00	-	-	-
25–29.9	1.10 (0.78–1.55)	0.59	-	-
30–34.9	1.31 (0.70–2.46)	0.40	-	-
≥35	0.67 (0.24–1.86)	0.44	-	-
Duration of infertility (years)				
<5	1.00	-	-	-
5–10	0.75 (0.57–1.00)	0.05	-	-
>10	0.45 (0.48–0.65)	0.16	-	-
Infertility diagnosis				
Female	1.00	-	-	-
Combined	1.33 (0.91–1.94)	0.14	-	-
PGT-M	2.47 (1.35–4.51)	0.00	-	-
Type of infertility				
Primary	1.00	-	-	-
Secondary	0.93 (0.71–1.22)	0.60	-	-
PGT-M	1.88 (1.12–3.16)	0.02	-	-
Parity				
0	1.00	-	-	-
1	0.95 (0.66–1.38)	0.80	-	-
2	1.23 (0.63–2.48)	0.52	-	-
3	0.59 (0.16–2.16)	0.43	-	-
Indication for PGT-A				
Male or female genetic abnormality	1.73 (1.03–2.92)	0.04	-	-
Previous child with genetic abnormality	0.84 (0.52–1.37)	0.48	-	-
Stem cell transplant from HLA matched	1.99 (0.49–7.99)	0.33	-	-
≥2 unexplained recurrent pregnancy losses	0.54 (0.28–1.03)	0.06	-	-
Advanced maternal age (≥35 years)	0.34 (0.17–0.68)	0.00	-	-
≥2 unsuccessful ART treatments	0.69 (0.44–1.09)	0.11	-	-
AFC				
<10	1.00	-	-	-
10–19	1.59 (1.19–2.13)	0.00	-	-
20–29	1.92 (0.85–4.35)	0.12	-	-
≥30	2.27 (0.56–9.16)	0.25	-	-

### Predictors of euploidy (univariate and multivariate analysis)

Univariate analysis (Table 2) showed that superior morphological parameters, including overall blastocyst grade, degree of expansion, ICM, and TE quality, were all significantly associated with higher euploidy rates (all *P* < 0.01). Maternal age also showed a strong correlation; compared with women <35 years, those aged 38–40 and ≥41 years had significantly lower odds of euploidy (OR: 0.32 and 0.12, respectively; *P* < 0.001). Other clinical factors, including male/female genetic abnormality (*P* = 0.04), PGT-M diagnosis (*P* = 0.00), and AFC 10–19 (*P* = 0.00), were also identified as significant univariate predictors. In contrast, BMI, duration of infertility, infertility type, and parity did not significantly affect euploidy rates.

After adjusting for confounders in the multivariate model, overall blastocyst grade, TE morphology, and maternal age remained significantly associated with euploidy. Excellent/Good embryos (OR:3.75, 95% CI: 2.56–5.50) and Average embryos (OR: 1.47, 95% CI: 1.00–2.14) maintained significantly higher euploidy odds compared to Poor embryos. Similarly, TE grades A (OR: 2.34) and B (OR:1.77) were significantly superior to grade C. The impact of age remained robust, with a sharp decline in euploidy odds from age 38 (*P* < 0.001), whereas no significant difference was observed in the 35–37 age group (*P* = 0.81).

### Pregnancy and ongoing implantation outcomes

**Figure 4:**
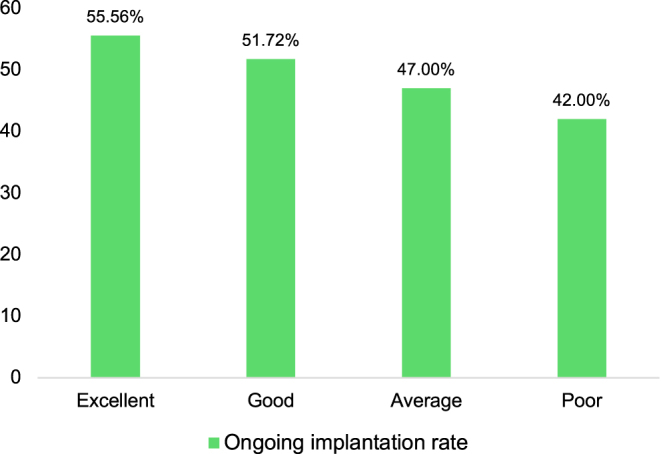
Ongoing implantation rate in different blastocyst morphologic grades.

Of 194 single euploid embryo transfers (SET) in 154 patients, the pregnancy rate was 53.09%, and the OIR was 42.27% (Table 3). The transferred blastocyst grades were 105 Excellent/Good, 47 Average, and 42 Poor, respectively. Morphology significantly impacted OIR:Excellent/Good grades achieved 52.38%, significantly higher than the Poor group’s 23.81% (*P* < 0.01; Fig. 4). Higher-quality blastocysts not only exhibited greater euploidy rates but also achieved better OIR outcomes compared to lower-quality blastocysts.

**Table 3:** Pregnancy outcomes following single euploid embryo transfer. Data are presented as *n* (%).

Parameters	Values
No. of euploid blastocysts transferred	194
Pregnancy	103 (53.09)
Biochemical pregnancy	12 (6.19)
Miscarriages	8 (4.12)
Ectopic pregnancy	1 (0.52)
Ongoing implantation rate	82 (42.27)

Logistic regression (Table 4) identified ICM grade as the strongest individual predictor of OIR. Grade A ICM exhibited a nearly 9-fold increase in implantation odds compared to grade C (OR: 8.80, 95% CI:2.02–38.25; *P* < 0.01). TE grades A and B also showed significantly higher OIR than grade C (*P* = 0.01). While expansion degree did not significantly affect OIR, maternal age remained critical; patients aged 38–40 and ≥41 years had significantly lower OIR than those <35 years (OR:0.14 and 0.11, respectively; *P* < 0.05).

**Table 4:** Logistic regression analysis of the association between blastocyst morphology, age group, and ongoing implantation rate.

Variable/value	OIR (%)	OR (95% CI)	*P* value
Blastocyst grade			
Excellent and good	52.38	3.52 (1.57–7.89)	0.00
Average	36.17	1.81 (0.72–4.58)	0.21
Poor	23.81	1.00	-
Degree of expansion			
5, 6	55.56	3.50 (0.88–13.93)	0.08
4	51.14	2.93 (0.97–8.83)	0.38
3	37.78	1.70 (0.52–5.56)	0.06
2	20.83	0.74 (0.18–3.04)	0.67
1	26.32	1.00	
ICM			
A	54.55	8.80 (2.02–38.25)	0.00
B	45.58	6.14 (1.76–21.42)	0.00
C	12.00	1.00	-
TE			
A	54.55	4.20 (1.33–13.26)	0.01
B	45.59	2.93 (1.25–6.90)	0.01
C	22.22	1.00	
Age group (years)			
<35	75.00	1.00	-
35–37	53.33	0.38 (0.07–1.99)	0.25
38–40	30.00	0.14 (0.03–0.76)	0.02
≥41	25.00	0.11 (0.02–0.79)	0.03

OIR, ongoing implantation rate.

## Discussion

The overall euploid rate observed in this study was 33.62%. Compared to previous studies, our study demonstrates a lower euploid rate. A retrospective cohort study in China reported an euploid rate of 40.4% (Li *et al.* 2022), while another study in Italy reported an euploid rate of 34.9% (Li *et al.* 2022). This discrepancy can be explained by the younger female age in their study, with a mean age of 32.04 ± 5.18 and 36.8 ± 4.24, respectively. In comparison, our study participants were 38.25 ± 2.88 years old.

Maternal age plays an integral role in determining the embryo’s euploidy rate. Our logistic regression analysis showed that maternal age greater than 38 years was significantly associated with a higher incidence of aneuploid embryos. These associations became more robust with more AMA. This finding is consistent with other studies (ACOG 2018, Dang *et al.* 2019, Borgstrom *et al.* 2021, Li *et al.* 2022, Zhang *et al.* 2022). While some other studies found the association begins at 35 years, our study supports the hypothesis that the aneuploid rate increases gradually at 35 years rather than sharply (ACOG 2018, Dang *et al.* 2019). This underscores the importance of counselling patients about age-related reproductive outcomes and incorporating PGT-A into treatment strategies for older patients to optimise outcomes.

Our study demonstrated a significant association between higher blastocyst quality and higher euploidy rates, consistent with previous findings that blastocyst morphology correlates strongly with euploid status. Overall, findings support the hypothesis that embryo morphology is a reliable predictor of euploidy rate (Irani *et al.* 2017, ACOG 2018, Wang *et al.* 2018, McDaniel *et al.* 2021, Li *et al.* 2022, Roos Kulmann *et al.* 2022, Dong *et al.* 2024). Specifically, the excellent-quality group exhibited a markedly higher euploidy rate of 65.38% compared to 54.74% in the good-quality group, 31.46% in the average-quality group, and 18.79% in the poor-quality group. This gradient underscores the predictive value of blastocyst morphology in determining chromosomal normalcy. However, the average-grade group showed euploidy rates comparable to the overall cohort average (33.62%), suggesting that morphology has weaker discriminatory value in intermediate-quality embryos and that its predictive strength is more pronounced at the extremes of the grading spectrum (Excellent/Good vs Poor). Nevertheless, these findings are consistent with those reported by other studies (Capalbo *et al.* 2014, Irani *et al.* 2017, ACOG 2018, Wang *et al.* 2018, Kim *et al.* 2019, Li *et al.* 2022, Roos Kulmann *et al.* 2022) that found a positive correlation between better blastocyst morphology and higher euploidy rates, suggesting that morphological assessment can be helpful in embryo selection (Irani *et al.* 2017, ACOG 2018, Li *et al.* 2022).

A pivotal debate in modern ART is whether blastocyst morphology retains prognostic value once chromosomal status is established. While some studies suggest that euploidy nullifies the predictive power of morphology (Capalbo *et al.* 2014, Irani *et al.* 2017), our data challenge this notion, particularly within an AMA cohort. We observed a striking divergence in OIR among euploid embryos: 52.38% for Excellent/Good grades versus only 23.81% for Poor grades, indicating that PGT-A reflects chromosomal competence but does not account for the embryo’s functional vitality. In our study population (mean female age 38.25 years), poor morphological grades likely reflect underlying cytoplasmic compromises, such as mitochondrial dysfunction or accumulation of oxidative stress, that are not detected by chromosomal screening alone.

Deconstructing the morphological components reveals that the ICM serves as the primary gatekeeper of viability in euploid transfers (Irani *et al.* 2017, ACOG 2018, Li *et al.* 2022, Sivanantham *et al.* 2022). Our multivariate analysis demonstrated that euploid embryos with grade C ICM yielded a markedly lower ongoing pregnancy rate of 12.00%, compared to 54.55% in grade A (OR: 8.80, 95% CI: 2.02–38.25; *P* < 0.01).

This finding suggests that, while aneuploidy causes implantation failure via genetic lethality, a sparsely cellular ICM (grade C) may fail due to insufficient cell mass to support proper lineage differentiation into the fetus. Interestingly, the degree of expansion did not show statistical significance (*P* > 0.05), suggesting that cellular mass is more predictive of implantation than blastocoel volume. Similarly, TE remained significantly associated with ongoing pregnancy outcomes among euploid embryos (grade A: 54.55% vs grade C: 22.22%), reinforcing that adequate TE cellularity is essential for the physical mechanics of invasion and placentation (ACOG 2018, Guo *et al.* 2020, Awadalla *et al.* 2021, Ozgur *et al.* 2021). Because TE morphology is a component of the composite blastocyst grading system, these findings should be interpreted as complementary contributions of individual morphological parameters rather than as statistically independent effects on overall blastocyst grade.

Furthermore, our results highlight the limitations of PGT-A in overcoming age-related fertility decline. Even after selecting for euploid embryos, the OIR dropped precipitously from 75.00% in women <35 years to 25.00% in women ≥41 years (OR: 0.11; *P* = 0.03). Although increasing maternal age is strongly associated with higher aneuploidy rates, emerging evidence suggests that the relationship between age and chromosomal abnormalities follows a *U*-shaped distribution across the reproductive lifespan and that distinct types of chromosomal errors may predominate at different ages (Gruhn *et al.* 2019, Wartosch *et al.* 2021, Verdyck *et al.* 2023, Elmerdahl Frederiksen *et al.* 2024). In addition, reproductive ageing involves multiple mechanisms beyond meiotic segregation errors alone, including mitochondrial dysfunction, epigenetic alterations, cohesin deterioration, and reduced endometrial receptivity (Vitagliano *et al.* 2023, Nunez-Calonge *et al.* 2024). These factors may contribute to the reduced implantation potential observed even among euploid embryos in older patients. Therefore, in women over 40 years of age, PGT-A should be considered primarily as a strategy to reduce miscarriage risk and shorten time-to-pregnancy rather than as a guarantee of implantation success comparable to younger patients.

These findings support the continued role of morphology-based assessment in embryo selection. However, morphology alone is insufficient for predicting chromosomal status, as a substantial proportion of embryos with Excellent (30.77%) or Good (41.61%) morphology were aneuploid. Previous studies have indicated that several aneuploid embryos, despite their high morphological scores, may not be ideal for selection (Capalbo *et al.* 2014, Minasi *et al.* 2016, Wang *et al.* 2018, Kim *et al.* 2019, McDaniel *et al.* 2021, Dong *et al.* 2024). These findings should also be incorporated into pre-test counselling. In addition, relying solely on traditional morphology-based selection to transfer can result in adverse reproductive outcomes. Therefore, combining traditional morphology evaluation with PGT-A provides a more comprehensive approach, improving the accuracy of embryo selection and reducing the risk of adverse reproductive outcomes, such as miscarriage.

The strengths of our study include a large sample size, embryo scoring by two experienced embryologists to reduce inter-observer variation, and comprehensive data on blastocyst parameters, age, and pregnancy outcomes. Nevertheless, it is essential to acknowledge certain limitations. The retrospective nature of this study may introduce selection biases, and the single-centre design limits the generalisability of the findings. Future research should incorporate an embryo morphokinetic study, utilising a deep learning model and a multi-centre cohort, to validate these results and explore the underlying mechanisms linking embryo quality to euploidy. Moreover, advancements in embryo assessment technologies could provide more precise and objective evaluations of embryo quality, potentially improving the predictive accuracy of euploidy rates.

## Conclusion

This study reinforces the significant positive correlation between blastocyst morphology and euploidy rates in IVF embryos undergoing PGT-A. Higher-quality blastocysts exhibited greater euploidy rates and achieved better ongoing implantation outcomes than lower-quality blastocysts. Euploidy is a prerequisite but not a guarantee of IVF success, especially in older women. Blastocyst morphology, particularly the quality of the ICM, remains a potent predictor of implantation. We recommend a hierarchical selection strategy prioritising high-grade euploid embryos to optimise outcomes in AMA patients.

## Declaration of interest

The authors declare that there is no conflict of interest that could be perceived as prejudicing the impartiality of the work reported.

## Funding

This research did not receive any specific grant from any funding agency in the public, commercial, or not-for-profit sector.

## Author contribution statement

All authors contributed to the study conception and design. CS, AS, and CSA performed material preparation, data collection, and analysis. CS wrote the first draft of the manuscript, edited by AS, and all authors commented on earlier versions. All authors read and approved the final manuscript.

## Ethics approval

This study protocol was approved by the Human Research Ethics Committee at the Faculty of Medicine, Ramathibodi Hospital, Mahidol University (MURA2023/222, approval date: 27 February 2023).

## Data availability

The data supporting this study’s findings are available from the authors upon reasonable request.
